# Influence of Parental and Some Demographic Characteristics on Overweight/Obesity Status among a Sample of Egyptian Children

**DOI:** 10.3889/oamjms.2016.088

**Published:** 2016-08-27

**Authors:** Nayera E. Hassan, Sahar A. El-Masry, Tarek Farid, Aya Khalil

**Affiliations:** 1 *Biological Anthropology Department, Cairo, Egypt*; 2 *Pediatric Department, Medical Research Division, National Research Centre, Cairo, Egypt (Affiliation ID 60014618)*

**Keywords:** Obesity, overweight, children, parental factors, residence

## Abstract

**BACKGROUND:**

Overweight/obesity is a multi-factorial problem, which results from rapidly changing social, economic, and physical environments that have led to an energy imbalance.

**AIM:**

To identify the association between childhood overweight/obesity and some socio-demographic risk factors, as parental age, body mass index (BMI), education and occupation, family size and residence (urban/rural).

**SUBJECTS AND METHODS:**

Cross-sectional study included 154 children of both sexes; aged 5-18 years; with their parents; one of them was working at the National Research Centre and from their relatives and neighbours. Data was collected about the child birth weight, family size, parental ages, education, occupation and place of residence. Anthropometric measurements including weight, height, and body mass index (BMI) of children and their parents were conducted.

**RESULTS:**

Obesity was detected among 19.5% of children (BMI > 95th percentile), 75.3% of their mothers and 49.6% of their fathers (BMI > 30 Kg/m^2). While overweight was present in 11.0% of the children (BMI > 85th- <95 percentile), 16.9% of their mothers and 36.5% of their fathers (BMI > 25-29.9 Kg/m^2). Child obesity was more prominent in urban than rural areas (21.3% versus 12.5%) and among housewives (22.8%) than among working mothers (16%, p < 0.016). Child overweight was more common in rural than urban areas (12.5% versus 10.7%) and among children with high father education (20%). Child BMI had significant positive correlations only with the child age, parental ages and BMIs, and family size. In spite of that, parental BMIs had significant positive correlations with each other and with family size, and significant negative correlations with maternal education and occupation and paternal education.

**CONCLUSION:**

Childhood obesity and overweight were more prominent in urban than rural areas, among children with non-working housewives mothers and highly educated fathers (college or above). Parental education and occupation had an indirect significant effect on child BMI through their significant effect on parental BMIs.

## Introduction

An important goal in addressing the global obesity epidemic is childhood obesity prevention [1]. In recent decades, the worldwide obesity epidemic is increasing at an alarming rate in childhood and can be observed in developing countries, which have shown an increase in the prevalence of childhood obesity [2].

Obesity is an established “risk factor” for several chronic diseases [3-5]. The World Health Organization [6] recognised obesity as a major public health epidemic.

Obesity is a multi-factorial problem, because of rapidly changing social, economic, and physical environments that have led to an energy imbalance in the population through a dramatic reduction in physical activity [7-8] and changes in dietary patterns [9].

Parental and familial characteristics are presumed to have an impact on offspring’s obesity status not only through shared genes but also through shared environments that determine nutrition and physical activity patterns early in life, as well as through the interaction of both [10].

Chaparroand Koupil [11] through their study on three generations of Swedish men and women; to investigate the impact of parental educational trajectories on their adult offspring’s overweight/obesity status; concluded that “Socioeconomic inequalities can have long-term consequences and impact the health of future generations. For overweight/obesity in their concurrent young cohorts, this inequality was not fully offset by an upward educational trajectory in their parent’s generation”.

In Egypt, as in other parts of the world, the obesity epidemic affects a growing number of children and adolescents. A study among female adolescents showed that 35 percent of the girls were overweight and 13 percent were obese. Overweight was more prevalent in urban than in rural girls [12]. In the report of diet, nutrition and prevention of chronic non-communicable diseases in Egyptian adolescents [13], it was found that about 20.5% of the adolescents were either overweight or obese with higher prevalence among urban than rural and females compared to males. In our previous study for assessment of the prevalence of metabolic syndrome among Egyptian school students suffering from obesity [14], the prevalence of obesity is 8.0% among 5798 Egyptian school students (26.4% were in the pre-pubertal period and 73.6% were in the pubertal period); however, the prevalence of overweight is 11%.

So, the purpose of current research is to estimate overweight and obesity prevalence and to identify factors, particularly parental risk factors as age, BMI, education, occupation, and demographic factors as family size and residence (urban/rural), that might be associated with overweight and obesity in a sample of children and adolescents aged 5–18 y to propose adapted action.

## Subjects and Methods

### Sample

This study was derived from a cross-sectional survey through a project funded by National Research Centre (NRC) Egypt, 2013-2016: entitled “Familiar Overweight and Obesity in Children and Adolescents: Diagnostic Clinical, Behavioral, Genetic and Biochemical Markers and Intervention” (10^th^ Research Plan of the NRC), after taking approval from Ethical Committee of NRC (Registration Number is 13/168).

A total number of 154 child of both sexes (77 males and 77 females) with their parents; one of them (mainly the mother) was working at the National Research Centre and from their relatives and neighbors; were chosen randomly from all categories of the workers to participate in the study after signing a written consent form of the Medical Ethical Committee of NRC. The age range of the children was between 5 and 18 years with a mean age was 10.83 ± 3.82. Participants were informed about the purpose of the study and their permission in the form of written consent was obtained.

### Methods

Trained interviewers collected detailed questionnaire from the mothers. Anthropometric measurements including weight, height, and body mass index (BMI) of the children and their parents were conducted.

### Detailed questionnaire

Data was collected by trained doctors about the child age in years; his/her birth weight in Kg., place of residence (urban/rural), family size (No. of persons in the family), parental (mothers and fathers) age, education and occupation. Parental education was classified into 3 grades: illiterate, precollege and college. Maternal occupation was graded as housewives or working mothers, while father’s occupation was graded as manual and non-manual workers.

### Anthropometric measurements

Weight was measured using a commercial scale (Seca Scale, Germany) with accuracy up to nearest 100 g. The subjects were asked to remove their footwear and wear minimal clothes before weighing them. Standing body height was measured, to the nearest 0.1 cm by using Holtain Stadiometer with the shoulder in a relaxed position and arms hanging freely and without shoes. The scales were recalibrated after each measurement following the recommendations of the International Biological Program [15]. Body Mass Index (BMI) was calculated as body weight in kilogrammes/height in metere^2. Children BMI percentile was calculated specific for age and sex based on the Egyptian Growth Reference Charts [16]. A child with BMI below 85^th^ percentile was considered healthy weight, with BMI between 85^th^ and95^th^ percentile overweight and those with BMI ≥ 95^th^ percentile obese. While mothers or fathers with BMI below 25 Kg/m^2 were considered healthy weight, with BMI ≥ 25-29.9 Kg/m^2 overweight and with BMI ≥ 30 Kg/m^2 were considered obese.

### Statistical analysis

Data were analysed using the Statistical Package for Social Sciences (SPSS/Windows Version 16, SPSS Inc., Chicago, IL, USA). Statistical significance was set at P < 0.05. Parametric data were expressed as mean ± SD, while the non-parametric data (qualitative) were expressed as frequency distribution: numbers and percentage of the total. Comparisons between the different non-parametric variables were analysed using Chi- square test. Spearman’s correlation test was used to examine the association between child and parental BMI with the different variables under study.

## Results

The distribution of the children and their parental BMI and some selected demographic variables was shown in Table 1. Obesity was detected among 19.5% of the children (their BMI > 95^th^ percentile), 75.3% of their mothers and 49.6% of their fathers (BMI > 30 Kg/m^2). While overweight was present in 11.0% of the children (their BMI >85^th^-<95^th^ percentile), 16.9% of their mothers and 36.5% of their fathers (BMI > 25-29.9 Kg/m^2).

**Table 1:** Descriptive characteristics of the study sample

Weight Status		
Child BMI [N (%)] (N = 154)	<85^th^ percentile	107 (69.5%)
	85^th^ to <95^th^ percentile	17 (11.0%)
	.> 95^th^ percentile	30 (19.5%)
Mother BMI [N (%)] (N = 154)	BMI < 25 Kg/m^2	12 (7.8%)
	BMI 25-29.9 Kg/m^2	26 (16.9%)
	BMI > 30 Kg/m^2	116 (75.3%)
Father BMI [N (%)] (N = 137)	BMI < 25 Kg/m^2	19 (13.9%)
	BMI 25-30 Kg/m^2	50 (36.5%)
	BMI > 30 Kg/m^2	68 (49.6%)

Child individual factors		
Child age years (Mean ±SD[Range])		10.83 ±3.82 [5.1-18]
Child sex	Boys [N(%)]	77 (50%)
	Girls (%)	77 (50%)
Birth weight (mean ± SD)		2.94 ±0.57

Family factors		
Maternal age years (mean ± SD)		38.92 ±8.48 [24-59]
Maternal Education	Illiterate	33 (21.7%
	Precollege	58 (38.2%)
	Collage	61 (40.1%)
Maternal Occupation	Housewife	95 (65.1%)
	Working	51 (34.9%)
Father age years (mean ± SD)		45.42 ±7.81 [30-60]
Father Education	Illiterate	23 (17.3%)
	Precollege	49 (36.8%)
	Collage	61 (45.9%)
Father Occupation135	Manual	66 (48.9%)
	Non-Manual	69 (51.1%)
Family size (N=153)		5.1 ±1.12 [2-8]
Residence	Rural	32 (20.8%)
	Urban	122 (79.2%)

The majority of the children (79.2%) were living in urban areas. The family size ranged between 2 up to 8 members in the family with a mean of 5.1 ± 1.12. Most of the mothers (65.1%) were non-working housewives, and 48.9% of the fathers were manual workers. College education or above was present in 40.1% of the mothers and 45.9% of the fathers.

The residence whether urban or rural was significantly related to the prevalence of obesity and overweight (Table 2), where obesity was more prominent in the urban areas (21.3% versus 12.5%) and overweight was more in rural areas (12.5% versus 10.7%). The maternal occupation had a significant effect on their childhood obesity, as obesity was more common among housewives (22.8%) than among working mothers (16%, p < 0.016).

**Table 2:** Frequency of child obesity and overweight (%) by age, sex and socioeconomic characteristics

Characteristics		N	Obesity (29)	Overweight (14)
Age	5-11	97	17 (17.5%)	10 (10.3%)
	12-18	57	13 (22.8%)	7 (12.3%)
	P value		0.456	0.467
Sex	Boys (%)	77	11 (14.3%)	10 (13%)
	Girls (%)	77	19 (24.7%)	7 (9.1%)
	P value		0.144	0.467
Maternal Education	Illiterate	33	9 (27.3%)	3 (9.1%)
	Precollege	54	12 (22.2%)	5 (9.3%)
	Collage	61	9 (14.8%)	8 (13.1%)
	P value		0.741	0.305
Maternal Occupation	Housewife	92	21 (22.8%)	7 (7.6%)
	Working	50	8 (16%)	8 (16%)
	P value		0.016*	0.796
Father Education	Illiterate	22	(40.9%)	1 (4.5%)
	Precollege	47	9 (19.1%)	4 (8.5%)
	Collage	60	8 (13.3%)	12 (20%)
	P value		0.962	0.003*
Father Occupation	Manual	62	13 (21%)	8 (12.9%)
	Non-Manual	69	14 (20.3%)	9 (13%)
	P value		0.847	0.808
Residence	Rural	32	4 (12.5%)	4 (12.5%)
	Urban	122	26 (21.3%)	13 (10.7%)
	P value		0.000**	0.029*

Father education had also a significant effect on their childhood overweight, which was the most prominent among children with father education of college or above (20%), and the least percentage was detected among children with illiterate fathers (4.5%, p < 0.003). However, there was the insignificant effect of the child age or sex, maternal education or father occupation on the prevalence of their children obesity and overweight.

Child BMI had significant positive correlations with the child age, parental ages and BMIs, and family size (Table 3). In addition, the parental education and occupation and the place of residence had insignificant correlations with child BMI.

**Table 3:** Correlation between child BMI and the other factors

Variables	Child BMI		Number
	r	p	
Child age	0.535**	0.000	145
Birth weight	0.125	0.136	143
Mother age	0.359**	0.000	145
Mother BMI	0.302**	0.000	148
Maternal Education	-0.136	0.099	148
Maternal Occupation	0.035	0.680	142
Father age	0.291**	0.001	120
Father BMI	0.227*	0.007	139
Father Education	-0.164	0.064	129
Father Occupation	-0.015	0.869	131
Residence U/R	-0.115	0.160	150
Family size	0.200*	0.015*	149

In spite of that, parental BMIs had significant positive correlations with each other and with family size and had significant negative correlations with maternal education and occupation and paternal education (Table 4). Moreover, Father BMI had significant negative correlations with father occupation and place of residence.

**Table 4:** Correlation between parental BMI and the other factors

	Mother BMI		Father BMI	
	r	p	r	p
Child age	0.089	0.281	0.095	0.267
Birth weight	0.137	0.105	-0.043	0.630
Mother age	0.063	0.443	0.075	0.382
Mother BMI	-	-	0.359	0.000**
Maternal Education	-0.262	0.001**	-0.254	0.002**
Maternal Occupation	-0.208	0.012*	-0.206	0.016
Father age	0.152	0.095	0.122	0.199
Father BMI	0.359	0.000**	-	-
Father Education	- 0.388	0.000**	-0.244	0.007**
Father Occupation	-0.089	0.304	-0.207	0.021*
Residence U/R	0.094	0.248	-0.260	0.002**
Family size	0.179	0.027*	0.196	0.020*

## Discussion

Several factors may play an important role in explaining child obesity, including parental influence, food prices, access to fast food, environment, and opportunities for physical activities, school nutrition policies, and advertising. Yet, such “root causes” cannot always explain excess variance within regions or racial groups [17]. In recent years, one additional explanation for the persistent increase in obesity levels – and one that has received considerable attention– is the effect of parental risk factors as age, BMI, education, employment, and demographic factors as family size and residence (urban/rural).

Current research aimed to investigate overweight and obesity prevalence and to identify factors, particularly parental risk factors and demographic factors as family size and residence (urban/rural), that might be associated with overweight and obesity in a sample of 154 child aged 5–18 y of both sexes, with their parents. Obesity was detected among 19.5% of the children, 75.3% of their mothers and 49.6% of their fathers. While overweight was present in 11.0 % of the children, 16.9% of their mothers and 36.5% of their fathers. The prevalence of overweight and obesity in this study was higher than that reported in previous Egyptian study conducted by Hafez et al. [18] who showed that the prevalence of overweight and obesity was 11% and 3.8%, respectively, among children attending government schools in Cairo. Hassan et al. [14]; in Giza; and El-Shafie and his colleagues [19]; in Dakahlia; found that the prevalence of overweight and obesity was 11.5 and 8.5%, respectively in primary school children.

This variation might be partially attributed to the difference in standard curves used for BMI Z-score classes. Nevertheless, this prevalence was higher than that reported in similar studies in UK and Brazil (8.7% and 4%) respectively [20].

Childhood obesity and overweight in the present study were more prominent in urban than rural areas, among children with non-working housewife mothers and highly educated fathers (college or above). In addition, they were positively correlated with child age, parental ages, BMIs and family size. Maternal and paternal BMIs recorded negative correlation with maternal education, occupation and paternal education; and positive correlation with each partner BMI and family size. Therefore, parental education and occupation had an indirect negative significant effect on child BMI through their significant effect on their parental BMIs.

Reviewing literatures for current results: as regards BMI-Z score classes, Bahbah et al. [21] in their study of obesity and overweight in primary school children living in Menoufia governorate; Menouf district; revealed that the incidence of obesity was higher among urban than among rural children; which coincides with our results; and children attending private schools and of high socioeconomic levels were more obese. This variation could be attributed to dietary variation between urban and rural children.

On the other hand, Koiralaa et al. [22] in their study on prevalence and factors associated with childhood overweight/obesity of private school children in Nepal found that children from families, having ≤ 2 siblings, upper-class family and advantaged ethnic group and children who were of larger birth weight (> 4.0 kg) had a greater likelihood of being overweight/obese.

Talat and El Shahat [23]; in Urban Sharkia Governorate; Egypt; concluded that risk factors for overweight and obesity were high in low level of parent education. The students of illiterate fathers and mothers had the highest incidence of obesity while the students of university fathers and mothers had the lowest incidence of obesity. The relation between the level of father’s & mother’s education and obesity was found to be significant. However, childhood obesity and overweight in the present study were more prominent in highly educated fathers (college or above).

Our study showed that parental education and occupation had an indirect negative significant effect on child BMI through their significant effect on their parental BMIs except father ’s education. As the highest prevalence of obesity was among children with low educated parents, since they are responsible for food selection for their children as well as their lifestyle activities. This agrees with several studies carried out in the developed countries which explain this association by the belief of low educated parents that overweight children are healthier than normal weight children. Therefore, they prefer high calories food which causes obesity in their children (Güven et al [24] in Turkey; Thibault et al [25] in France). Like our study, most studies carried out in developing countries revealed that the highest prevalence of obesity was among children with high educated parents due to parental style with low energy expenditure [(Matijasevich et al [20] in Brazil; Andegiorgish et al [26] in China).

In addition, there was indirect significant association between obesity and *fathers’ occupation* since the highest prevalence of obesity was among students of unskilled workers fathers while the lowest percentage was among students of professional fathers. This agrees with several Turkish and Australian studies which showed that “fathers’ socioeconomic status has an impact on the stable household habits, dietary values and physical activity” [27-28].

Results revealed an insignificant association between obesity and *working status of the mother* where obesity is significantly related to non-working housewife mothers; as obesity occurs due to unhealthy eating habits and sedentary lifestyle rather than working status of the mother. This goes parallel with Güven et al. [24], in Turkey. However, other studies in developing countries found a significant association between obesity and working status of the mother as the child is more likely to be overweight if his mother works more hours per week during childhood that impede young children’s access to healthy foods and physical activity [25, 29].

In conclusion, childhood obesity and overweight were more prominent in rural than urban areas, among children with non-working housewives mothers and highly educated fathers (college or above). Parental education and occupation had an indirect significant effect on child BMI through their significant effect on their parental BMIs.
